# Suicide risk in people with tuberculosis in Republic of Korea: a nationwide cohort, 2012–2021

**DOI:** 10.1186/s40249-026-01424-x

**Published:** 2026-03-23

**Authors:** Chiwook Chung, Seung Won Lee, Dawoon Jeong, Hongjo Choi, Hojoon Sohn, Young Ae Kang

**Affiliations:** 1 Division of Pulmonary, Allergy and Critical Care Medicine, Department of Internal Medicine, Hallym University Dongtan Sacred Heart Hospital, Hallym University College of Medicine, Hwaseong, Republic of Korea; 2 Institute for Immunology and Immunological Disease, Yonsei University College of Medicine, Seoul, Republic of Korea; 3 Department of Preventive Medicine, Seoul National University, Seoul, Republic of Korea; 4 Division of Health Policy and Management, Korea University College of Health Science, Seoul, Republic of Korea; 5 Division of Pulmonary and Critical Care Medicine, Department of Internal Medicine, Severance Hospital, Yonsei University College of Medicine, Seoul, Republic of Korea; 6 Institute for Innovation in Digital Healthcare, Yonsei University, 50-1 Yonsei-Ro, Seodaemun-Gu, Seoul, 03722 Republic of Korea

**Keywords:** Tuberculosis, Suicide, Mental health, Cause of death, Republic of Korea

## Abstract

**Background:**

Many individuals with tuberculosis (TB) experience various psychological problems after TB diagnosis, and they may also have suicidal ideations and suicidal attempts associated with TB-related depression or stigma. Until now, little is known about suicide rates among individuals with TB. We aimed to investigate the trends, characteristics, and contributing factors of suicide deaths among individuals with TB, using a Republic of Korea nationwide cohort.

**Methods:**

This was a retrospective nationwide cohort study using data of the K-TB-N cohort. We analyzed 310,194 individuals with TB registered in the Korean National Tuberculosis Surveillance System between 2012 and 2021. Suicidal deaths were identified using the International Classification of Diseases 10th revision codes (X60–X84) from the Statistics Korea database. Participants were followed up until death, censoring, or December 2022. We calculated suicide incidence rates, examined temporal distribution of suicide occurrence following TB diagnosis using Poisson regression, and used multivariate Cox proportional hazards models, including age-stratified analyses, to identify factors associated with suicide.

**Results:**

Altogether, 1314 (0.42%) died by suicide [1.6% of all deaths (1314/80,323)]. Men and individuals aged ≥ 60 years accounted for 76.9% and 56.1% of suicide deaths, respectively. Overall suicide incidence was 0.77/1000 person-years, more than twice as high in men compared to that in women (1.03 vs. 0.42/1000 person-years), and increased with age (from 0.32 in 20–29 years to 1.59 in ≥ 80 years/1000 person-years). Suicide incidence within one year after TB diagnosis was 2.04/1000 person-years. Notably, 31.3% of the suicides occurred during TB treatment. The incidence of suicide was highest immediately after TB diagnosis and tended to decrease over time. Male sex, older age, pulmonary TB, sputum smear positivity, comorbidities, and mental disorders were significantly associated with higher suicide risk. The risk factors for suicide showed different patterns across age groups (younger adults: pre-existing mental health conditions; middle-aged adults: low income and infectiousness; and older adults: comorbidities and disability).

**Conclusions:**

Suicide is a notable cause of death among individuals with TB in Republic of Korea, particularly early after diagnosis. The factors contributing to suicide risk differed depending on sex and age. Routine mental health screening and early targeted psychosocial intervention strategies should be integrated into national TB care programs to reduce preventable deaths.

**Graphical abstract:**

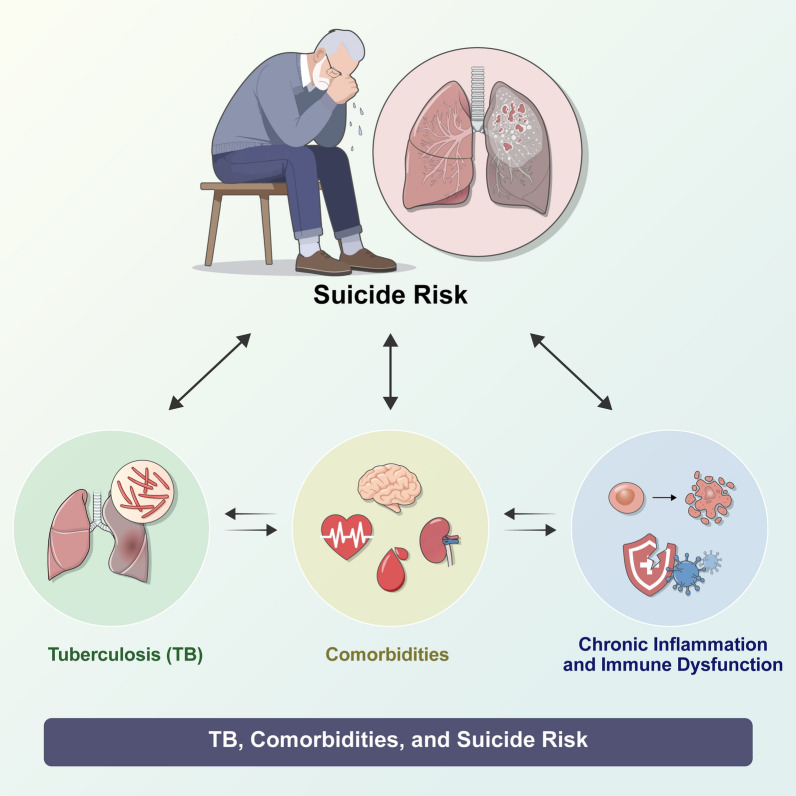

**Supplementary Information:**

The online version contains supplementary material available at 10.1186/s40249-026-01424-x.

## Background

Tuberculosis (TB) remains a significant global health concern, particularly in resource-limited high-burden countries [1]. As per the World Health Organization (WHO), there were approximately 10.8 million new TB cases and 1.25 million TB-related deaths in 2023 [1]. Beyond the physical morbidity and mortality associated with TB, there is growing recognition of the mental health and social burdens experienced by individuals diagnosed with TB [2]. These include stigma, social isolation, economic burden accompanying prolonged treatment, and comorbidities such as depression and anxiety [3–5]. More than 30% of patients with TB experienced depression [5]. The presence of these mental health issues not only impairs quality of life and treatment adherence but also increases the risk of suicidal behaviors among affected individuals [6, 7].

Despite substantial progress in national TB control programs, Republic of Korea has the highest TB incidence (39.8/100,000 population in 2022) and mortality (3.8/100,000 population in 2022) among the Organization for Economic Co-operation and Development (OECD) countries [8, 9]. In addition, suicide is a major public health issue in Republic of Korea, with a suicide rate of approximately 27.3/100,000 population in 2023 (the 5th leading cause of death), significantly exceeding the OECD average (approximately 11/100,000 population in 2020) [10, 11]. Mental health challenges in patients with TB may be exacerbated by the chronic nature of the disease, adverse drug effects, socioeconomic vulnerabilities, stigma associated with infectious diseases, and exacerbation of comorbidities [3, 12]. Thus, TB-related neuroendocrinological dysfunction, stigma, depression, social exclusion, and economic hardship may have collectively contributed to increase suicide risk [3, 13–16].

While limited, existing studies from countries such as South Africa and Ethiopia suggest elevated suicide risk among TB patients, particularly during early treatment stages [17, 18]. A recent meta-analysis reported that the pooled prevalence of current suicidal ideations and suicidal attempts within the last year among individuals with TB was 8.5% [95% confidence interval (*CI*) 5.8–12.3%] and 3.1% (95% *CI* 2.2–4.5%), respectively [7]. Although this analysis included nine studies and 8770 participants, several methodological limitations were noted. Most of the included studies employed a cross-sectional design and had relatively small sample sizes, and few studies provided data on suicide deaths, highlighting the need for additional epidemiological evidence [7]. Moreover, this analysis also highlighted the lack of large-scale longitudinal studies assessing actual suicide mortality (not just ideation or attempts) among individuals with TB, particularly in the Western Pacific or high-income Asian countries [7].

Understanding the burden and risk factors for suicide among individuals with TB is essential for informing integrated care strategies that incorporate mental health support into TB control programs. The primary objective of this study was to estimate the incidence of suicide and identify clinical and sociodemographic factors associated with suicide risk among individuals with TB, thereby providing evidence to inform integrated TB–mental health care strategies. Therefore, we explored the incidence and risk factors for suicide among individuals with TB using the Republic of Korea national TB cohort (K-TB-N cohort) [19]. To overcome the limitations of previous studies (e.g. a cross-sectional design and small sample sizes), we 1) constructed a nationwide retrospective cohort; 2) identified suicide deaths using national cause-of-death statistics; and 3) explored the incidence, demographics, disease-related factors, and comorbidities associated with suicide risk among patients with TB.

## Methods

### Data source

**Fig. 1:**
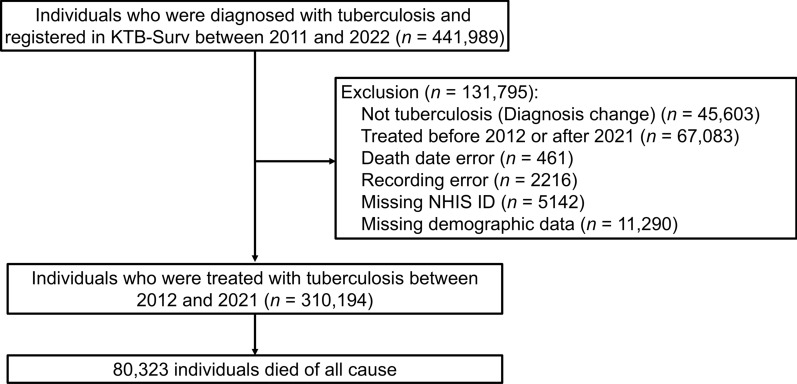
Study flowchart for participants inclusion process. KTB-Surv, Korean Tuberculosis Surveillance System

The K-TB-N cohort was constructed by linking three databases: 1) the Korean National Tuberculosis Surveillance System (KTB-Surv) established by the Korea Disease Control and Prevention Agency, containing TB notification data for those registered between 2011 and 2022 [20]; 2) the National Health Information Database (NHID), established by the National Health Insurance Service (NHIS) [21]; and 3) the Statistics Korea database on cause-of-death statistics [19]. The KTB-Surv database contains individual patient data including age, sex, nationality, TB notification date, TB lesion (pulmonary or extrapulmonary), sputum acid-fast bacillus (AFB) smear, previous TB history, and drug susceptibility test results. The NHID database contains additional patient data, including 1) socio-demographic data (e.g., age, sex, residential region, household income, and death), 2) disease diagnosis (according to the International Classification of Diseases 10th revision, ICD-10), 3) medications (such as name, quantity, and duration), 4) health services types used (e.g., inpatient procedures, prescriptions, and operations), and 5) healthcare provider information (location, level, and type of healthcare provider). The Statistics Korea database provides information on underlying causes of death. All databases were linked using deterministic matching through unique national identification numbers, ensuring exact one-to-one correspondence between individuals across datasets. As shown in Fig. 1, individuals without valid NHIS identifiers or with missing linkage keys were excluded from the analysis to ensure accurate matching across datasets.

### Study population

This was a nationwide retrospective cohort study of individuals with TB, conducted from May 2023 to April 2025. Figure 1 illustrates the participant inclusion process. The KTB-Surv database identified 441,989 individuals with TB who were registered and treated between 2011 and 2022. Among them, we excluded individuals with 1) change in the diagnosis (not TB, *n* = 45,603), 2) treatment before 2012 or after 2021 (2011: because of incompleteness of KTB-Surv database, and 2022: owing to ongoing treatment and unconfirmed treatment outcomes, *n* = 67,083), 3) death date error (*n* = 461), 4) recording error (*n* = 2216), 5) missing NHIS ID (*n* = 5142), and 6) missing demographic data (*n* = 11,290). Consequently, the remaining 310,194 individuals with TB who were registered and treated between 2012 and 2021 were included in this study. All participants were followed up until December 2022. The follow-up ended at death (including suicide, the study outcome) or censoring. The overall mean follow-up duration was 5.5 ± 3.3 years.

### Measurements

The primary outcome of this study was suicide death identified by the cause of death as ICD-10 codes X60–84 (intentional self-harm) [6]. We identified the cause of death from the national mortality statistic of Statistics Korea [22].

Data of demographic, socioeconomic, TB-related, comorbid, and mental disorder factors were collected from the KTB-Surv and the NHID. The demographic variables included age and sex. Socioeconomic variables included residential region, household income, and disability status. The residential regions were classified into provinces and metropolitan areas. Household income levels were categorized into quintiles (Q1 = lowest; Q5 = highest) based on subscribers’ annual national health insurance premiums. Medical aid beneficiaries (lowest 3%) were categorized as Q0. TB-related variables included TB lesions (pulmonary vs. extrapulmonary), drug resistance (either isoniazid or rifampin/multidrug resistance), previous TB history, and sputum AFB smear positivity.

Comorbidities were evaluated using the Charlson comorbidity index (CCI) score [23] and data of cardiovascular disease, cerebrovascular disease, dementia, chronic obstructive pulmonary disease, rheumatic disease, peptic ulcer, chronic liver disease, diabetes, hemiplegia, chronic kidney disease, malignancy (either solid cancer or hematologic malignancy), and living with human immunodeficiency virus (HIV). Mental disorders included depression, anxiety, bipolar disorder, affective disorder, schizophrenia, alcohol use disorder, and substance use disorder. Comorbidities and mental disorders were identified using insurance claims of relevant ICD-10 codes within one year prior to the TB index date (Supplementary Table 1).

### Statistical analysis

Continuous variables are presented as mean with standard deviation if normally distributed. Categorical variables are presented as proportion/percentages. Student t-test (for normally distributed variables) or the Mann–Whitney U test was used to compare continuous variables, whereas the chi-squared or Fisher exact tests were used to compare categorical variables. The incidence rate of suicide was calculated as the ratio of the number of suicide deaths to the number of person-years (PY) at risk (per 1000 PY). Poisson regression was used to assess changes in suicide incidence according to time elapsed since TB diagnosis. The Kaplan–Meier survival curve was used to describe the cumulative incidence of suicide. To identify factors associated with suicide, a Cox proportional hazards model with non-suicide death treated as a competing event was used. Covariates included sex, age, residential region, household income, presence of disabilities, type of TB lesions, drug resistance, previous TB history, sputum AFB smears, comorbidities, and mental disorders. Demographic and TB-related variables were included in the multivariate analysis, regardless of the *P* values in the univariate analysis, while other variables showing statistical significance (*P* < 0.05) in the univariate analysis were also entered into the multivariate model. Multicollinearity among variables was assessed using the Variance Inflation Factor. As for comorbidities, Model 1 included CCI scores, whereas Model 2 included individual disease categories. Sex and age (< 40, 40–69, and > 70 years) stratified analyses were conducted to identify specific risk factors for suicide. In addition, stratified analyses were conducted according to the TB treatment phase (during vs. after TB treatment) to explore potential differences in risk factors across these periods. All *P* values were two-tailed, and *P* values < 0.05 were considered statistically significant. Statistical analyses were performed using the SAS Enterprise Guide 7.1 (SAS Institute Inc., Cary, NC, USA).

## Results

### General characteristics of participants

Among the 80,323 all-cause deaths, 1314 were identified as suicidal deaths. Table 1 presents the general characteristics of the study population according to vital status and cause of death. The mean age of the population was 57.8 ± 20.0 years, with men comprising 59.1%. Regarding the demographic characteristics, the suicidal death group had the highest proportion of men (76.9%), whereas the mean age in the non-suicidal death group was the highest (74.3 ± 13.0 years). The proportion of those with AFB smear positivity was higher in the suicide group (37.7%) and non-suicide groups (37.3%) than in the survivors (27.0%). Regarding comorbidities, both the CCI score and the proportions of individual comorbid diseases increased in the order of survivors, suicide group, and non-suicide group. Mental disorders were the least frequent in the survivor group and most frequent in the suicidal death group. In the comparison by sex, a higher proportion of older adults was observed in women, whereas there was no notable difference in comorbidities and mental disorders between sexes (Supplementary Table 2).

**Table 1:** General characteristics of individuals with tuberculosis by vital status and cause of death

*N*	Total		Survivor		Suicide death		Non-suicide death	
	310,194 (100%)		229,871 (74.11%)		1314 (0.42%)		79,009 (25.47%)	
	*n*	%	*n*	%	*n*	%	*n*	%
Male sex	183,277	59.08	132,273	57.54	1010	76.86	49,994	63.28
Age, years	57.8 ± 20.0		52.1 ± 18.9		61.5 ± 17.9		74.3 ± 13.0	
0–19	7686	2.48	7615	3.31	15	1.14	56	0.07
20–29	27,687	8.93	27,407	11.92	65	4.95	215	0.27
30–39	30,316	9.77	29,491	12.83	94	7.15	731	0.93
40–49	39,365	12.69	36,122	15.71	149	11.34	3094	3.92
50–59	52,539	16.94	44,740	19.46	254	19.33	7545	9.55
60–69	46,192	14.89	34,980	15.22	205	15.60	11,007	13.93
70–79	56,695	18.28	32,543	14.16	305	23.21	23,847	30.18
≥ 80	49,714	16.03	16,973	7.38	227	17.28	32,514	41.15
Region (Province)	176,247	56.82	126,987	55.24	784	59.67	48,476	61.36
Low income (Q0)	25,111	8.10	13,510	5.88	131	9.97	11,470	14.52
Low income (Q1)	51,756	16.69	39,027	16.98	221	16.82	12,508	15.83
Disabilities	42,645	13.75	22,771	9.91	252	19.18	19,622	24.84
Pulmonary TB	246,326	79.41	180,365	78.46	1121	85.31	64,840	82.07
Drug resistance	13,889	4.48	10,616	4.62	64	4.87	3209	4.06
Previous TB history	32,856	10.59	23,087	10.04	193	14.69	9576	12.12
Positive AFB smear	92,039	29.67	62,050	26.99	495	37.67	29,494	37.33
CCI score	2.2 ± 1.9		1.7 ± 1.7		2.5 ± 2.0		3.4 ± 2.1	
Cardiovascular disease	63,974	20.62	34,318	14.93	320	24.35	29,336	37.13
Cerebrovascular disease	39,604	12.77	17,902	7.79	182	13.85	21,520	27.24
Dementia	28,680	9.25	9050	3.94	77	5.86	19,553	24.75
COPD	136,523	44.01	92,229	40.12	645	49.09	43,649	55.25
Rheumatic disease	13,332	4.30	8919	3.88	77	5.86	4336	5.49
Peptic ulcer	82,073	26.46	54,403	23.67	421	32.04	27,249	34.49
Chronic liver disease	92,296	29.75	60,694	26.40	486	36.99	31,116	39.38
Diabetes	91,933	29.64	54,282	23.61	460	35.01	37,191	47.07
Hemiplegia	5989	1.93	1976	0.86	27	2.05	3986	5.04
Chronic kidney disease	13,423	4.33	5548	2.41	53	4.03	7822	9.90
Malignancy	35,985	11.60	19,584	8.52	168	12.79	16,233	20.55
Living with HIV	594	0.19	456	0.20	7	0.53	131	0.17
Depression	37,076	11.95	20,240	8.80	310	23.59	16,526	20.92
Anxiety disorder	49,944	16.10	29,848	12.98	352	26.79	19,744	24.99
Bipolar disorder	11,659	3.76	4197	1.83	71	5.40	7391	9.35
Affective disorder	2009	0.65	1212	0.53	27	2.05	770	0.97
Schizophrenia	7112	2.29	3403	1.48	64	4.87	3645	4.61
Alcohol use disorder	5755	1.86	3123	1.36	54	4.11	2578	3.26
Substance use disorder	334	0.11	191	0.08	6	0.46	137	0.17

*TB* tuberculosis; *AFB* acid-fast bacilli; *CCI* Charlson comorbidity index; *COPD* chronic obstructive pulmonary disease; *HIV* human immunodeficiency virus

### Incidence and characteristics of suicide by sex and age group

Table 2 presents the distribution of suicidal deaths stratified by sex, age, and year of diagnosis. The incidence rate of suicide was 0.77/1000 PY, which was more than twice as high in men compared to that in women (1.03 vs. 0.42/1000 PY, respectively). The incidence rate also increased with age (0–19 years: 0.25/1000 PY vs. ≥ 80 years: 1.59/1000 PY). The incidence rate of suicide within one year after TB diagnosis was 2.04/1000 PY. Although a decreasing trend in the number of suicidal deaths within one year after TB diagnosis was observed longitudinally (2012: 93 cases vs. 2021: 33 cases), the incidence rate remained relatively stable throughout the study period owing to a decreasing trend in the total number of TB cases.

**Table 2:** Distribution of suicide deaths among tuberculosis patients stratified by sex, age group, and year of diagnosis

	*n*	Total suicide deaths			Suicide within 1 year after TB diagnosis		
		Duration (PY)	Suicide	IR (per 1000 PY) with 95% *CI*	Duration (PY)	Suicide	IR (per 1000 PY) with 95% *CI*
Total	310,194	1,704,159.42	1314	0.77 (0.73–0.81)	283,179.53	579	2.04 (1.88–2.22)
Male	183,277	985,358.06	1010	1.03 (0.96–1.09)	166,406.75	443	2.66 (2.42–2.92)
Female	126,917	718,801.36	304	0.42 (0.38–0.47)	116,772.78	136	1.16 (0.98–1.38)
Age, years							
0–19	7686	59,622.25	15	0.25 (0.15–0.42)	7664.48	1	0.13 (0–0.73)
20–29	27,687	202,672.33	65	0.32 (0.25–0.41)	27,608.59	16	0.58 (0.33–0.94)
30–39	30,316	215,629.77	94	0.44 (0.36–0.53)	30,118.45	23	0.76 (0.48–1.15)
40–49	39,365	262,825.92	149	0.57 (0.48–0.67)	38,554.90	51	1.32 (0.98–1.74)
50–59	52,539	317,532.07	254	0.80 (0.71–0.90)	50,486.38	98	1.94 (1.58–2.37)
60–69	46,192	242,854.20	205	0.84 (0.74–0.97)	43,024.17	96	2.23 (1.81–2.72)
70–79	56,695	260,258.87	305	1.17 (1.05–1.31)	49,370.00	152	3.08 (2.61–3.61)
≥ 80	49,714	142,764.01	227	1.59 (1.40–1.81)	36,352.54	142	3.91 (3.29–4.60)
Index Year							
2012	42,611	367,354.98	269	0.73 (0.65–0.83)	39,970.74	93	2.33 (1.88–2.85)
2013	38,306	300,857.12	205	0.68 (0.59–0.78)	35,829.07	68	1.90 (1.47–2.41)
2014	37,164	260,186.72	208	0.80 (0.70–0.92)	34,424.65	66	1.92 (1.48–2.44)
2015	34,075	210,945.41	141	0.67 (0.57–0.79)	31,339.98	63	2.01 (1.54–2.57)
2016	32,939	176,057.42	126	0.72 (0.60–0.85)	30,004.57	52	1.73 (1.29–2.27)
2017	30,274	137,942.87	101	0.73 (0.60–0.89)	27,474.00	47	1.71 (1.26–2.27)
2018	28,450	106,094.65	101	0.95 (0.78–1.16)	25,552.87	58	2.27 (1.72–2.93)
2019	25,570	75,030.00	78	1.04 (0.83–1.30)	22,834.06	59	2.58 (1.97–3.33)
2020	21,210	44,467.47	52	1.17 (0.89–1.53)	18,650.02	40	2.14 (1.53–2.92)
2021	19,595	25,222.77	33	1.31 (0.93–1.84)	17,099.57	33	1.93 (1.33–2.71)

*PY* person-year; *IR* incidence rate; *CI* confidence interval

### Temporal distribution of suicide after TB diagnosis

**Fig. 2:**
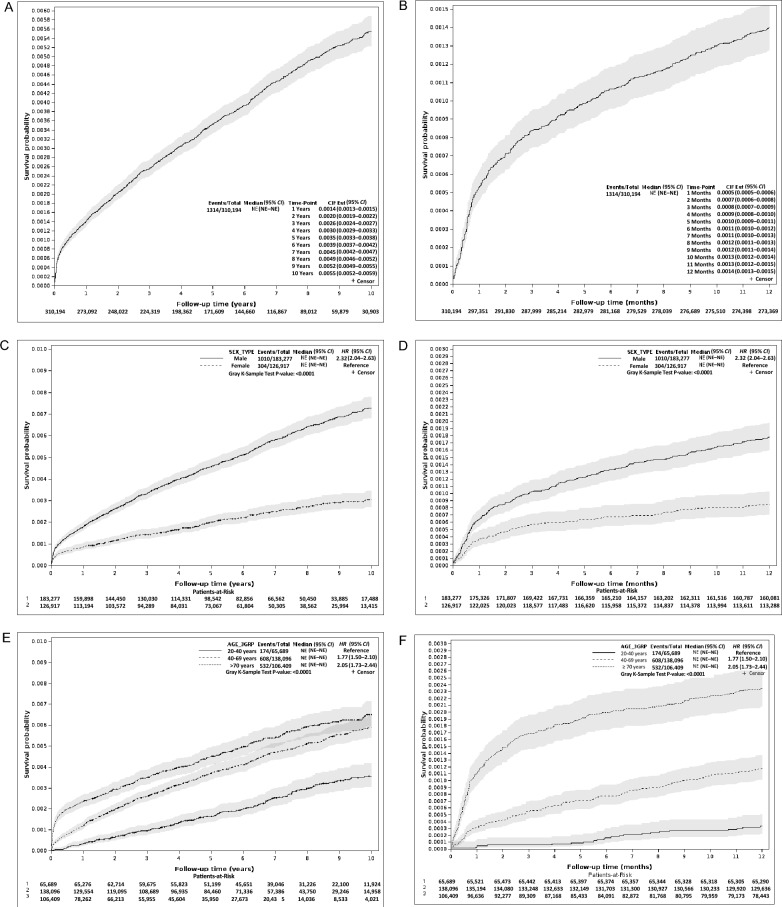
Cumulative incidence of suicide death since tuberculosis diagnosis stratified by sex and age, plotted using the Kaplan–Meier survival curve. **A** Total study period, **B** Within one year after tuberculosis diagnosis, **C** Total study period and stratified by sex, **D** Within one year after tuberculosis diagnosis and stratified by sex, **E** Total study period and stratified by age, and **F** Within one year after tuberculosis diagnosis and stratified by age

Table 3 shows the distribution of suicidal deaths according to the time elapsed since TB diagnosis. Approximately one-third (31.3%, 411/1314; male: 311, famale: 100) of the suicides occurred during TB treatment. In particular, 12.6% (166/1314) and 19.9% (261/1314) of the cases occurred within 30 and 90 days of TB diagnosis, respectively. Thereafter, the incidence of suicide gradually decreased over time (*P* < 0.001, Fig. 2, Supplementary Fig. 1).

**Table 3:** Numbers of suicide deaths by time interval since tuberculosis diagnosis stratified by sex

Time to suicide, days	Total	Index year									
		2012	2013	2014	2015	2016	2017	2018	2019	2020	2021
Total											
0–30	166	28	14	18	13	17	11	14	24	10	17
31–60	55	8	8	4	4	6	3	8	6	6	2
61–90	40	8	2	2	3	3	4	8	4	3	3
91–120	25	3	4	4	4	4	2	2	0	1	1
121–150	20	5	1	3	1	0	2	2	1	2	3
151–180	24	4	5	0	4	2	0	3	2	1	3
181–270	57	8	9	8	11	4	3	2	7	4	1
271–360	47	8	9	9	5	6	3	2	2	3	0
≥ 361	880	197	153	160	96	84	73	60	32	22	3
Male											
0–30	119	25	7	13	10	12	5	10	18	6	13
31–60	41	7	4	1	3	5	3	7	4	5	2
61–90	28	6	2	2	3	2	1	5	4	2	1
91–120	22	1	4	3	4	4	2	2	0	1	1
121–150	15	4	1	1	0	0	2	1	1	2	3
151–180	19	2	4	0	3	2	0	2	2	1	3
181–270	44	5	8	8	10	1	2	1	6	3	0
271–360	38	6	5	8	4	6	3	1	2	3	0
≥ 361	684	148	121	116	80	66	61	48	23	18	3
Female											
0–30	47	3	7	5	3	5	6	4	6	4	4
31–60	14	1	4	3	1	1	0	1	2	1	0
61–90	12	2	0	0	0	1	3	3	0	1	2
91–120	3	2	0	1	0	0	0	0	0	0	0
121–150	5	1	0	2	1	0	0	1	0	0	0
151–180	5	2	1	0	1	0	0	1	0	0	0
181–270	13	3	1	0	1	3	1	1	1	1	1
271–360	9	2	4	1	1	0	0	1	0	0	0
≥ 361	196	49	32	44	16	18	12	12	9	4	0

### Contributing factors to suicide among individuals with TB

Our multivariate analysis models identified several significant factors contributing to suicidal death. Male sex, older age, low income, disability, pulmonary TB, and AFB smear positivity were significant factors contributing to suicide. Among comorbidities, a higher CCI score, rheumatic disease, chronic liver disease, and living with HIV were factors significantly contributing to suicide death. Among mental disorders, depression, anxiety disorder, affective disorder, and schizophrenia were significant contributing factors to suicide death (Table 4).

**Table 4:** Estimated hazard ratios for demographic, tuberculosis-related, and comorbid risk factors contributing to suicide death after tuberculosis diagnosis

	Univariate analysis			Multivariate analysis Model 1			Multivariate analysis Model 2		
	*HR*	95% *CI*		a*HR*	95% *CI*		a*HR*	95% *CI*	
Male sex	2.398	2.109	2.726	2.675	2.342	3.055	2.669	2.335	3.050
Age, years									
0–19	Ref			Ref			Ref		
20–29	1.261	0.720	2.211	1.250	0.713	2.193	1.236	0.705	2.169
30–39	1.707	0.990	2.943	1.565	0.906	2.702	1.536	0.889	2.652
40–49	2.199	1.293	3.738	1.705	1.000	2.905	1.658	0.973	2.827
50–59	3.053	1.814	5.138	2.086	1.235	3.526	2.033	1.202	3.438
60–69	3.145	1.862	5.313	2.066	1.216	3.511	2.036	1.197	3.463
70–79	4.276	2.546	7.181	2.919	1.722	4.951	2.931	1.727	4.975
≥ 80	5.278	3.127	8.907	3.938	2.307	6.724	4.051	2.371	6.920
Region (Province)	1.184	1.060	1.322	1.077	0.964	1.204	1.078	0.964	1.204
Low income (Q0)	1.556	1.265	1.915	1.139	0.916	1.415	1.133	0.911	1.408
Low income (Q1)	1.076	0.903	1.283	1.248	1.044	1.493	1.246	1.041	1.490
Disabilities	1.868	1.628	2.143	1.237	1.070	1.431	1.264	1.091	1.463
Pulmonary TB	1.510	1.296	1.759	1.231	1.025	1.479	1.241	1.033	1.492
Drug resistance	1.201	0.934	1.545	1.078	0.836	1.388	1.076	0.835	1.387
Previous TB history	1.435	1.232	1.673	1.167	0.999	1.362	1.171	1.003	1.367
Positive AFB smear	1.435	1.278	1.611	1.306	1.159	1.472	1.310	1.162	1.476
CCI score	1.200	1.169	1.233	1.045	1.011	1.080			
Cardiovascular disease	1.718	1.513	1.950				1.080	0.938	1.243
Cerebrovascular disease	1.577	1.348	1.845				0.846	0.711	1.007
Dementia	1.151	0.913	1.451						
COPD	1.372	1.231	1.529				1.016	0.906	1.139
Rheumatic disease	1.534	1.219	1.932				1.279	1.012	1.616
Peptic ulcer	1.418	1.263	1.592				1.066	0.944	1.205
Chronic liver disease	1.593	1.424	1.782				1.178	1.045	1.328
Diabetes	1.633	1.457	1.829				1.031	0.910	1.167
Hemiplegia	1.731	1.182	2.536				1.073	0.721	1.598
Chronic kidney disease	1.465	1.112	1.929				0.963	0.726	1.279
Malignancy	1.464	1.244	1.721				1.135	0.961	1.340
Living with HIV	2.703	1.286	5.681				2.392	1.134	5.044
Depression	2.854	2.512	3.243	1.859	1.600	2.159	1.885	1.623	2.190
Anxiety disorder	2.228	1.971	2.517	1.444	1.255	1.661	1.441	1.252	1.658
Bipolar disorder	2.530	1.990	3.216	1.084	0.834	1.408	1.113	0.856	1.447
Affective disorder	3.566	2.436	5.220	2.004	1.361	2.952	2.025	1.375	2.984
Schizophrenia	2.891	2.249	3.718	1.602	1.217	2.108	1.610	1.223	2.120
Alcohol use disorder	2.509	1.910	3.294	1.173	0.876	1.570	1.136	0.847	1.523
Substance use disorder	4.797	2.151	10.698	2.296	1.024	5.146	2.234	0.997	5.010

*HR* hazard ratio; *aHR* adjusted hazard ratio; *CI*, confidence interval; *Ref.* reference; *TB* tuberculosis; *AFB* acid-fast bacilli; *CCI* Charlson comorbidity index; *COPD* chronic obstructive pulmonary disease; *HIV* human immunodeficiency virus

In the analysis stratified by sex, older age was the most significant contributing factor among men, whereas it had no contribution in women (Supplementary Tables 3 and 4). In the younger age group (< 40 years), depression and schizophrenia were the only mental disorders that were contributing factors to suicide. In contrast, low household income status and AFB smear positivity were significant contributing factors in the 40–69 years age group (economically active population) in addition to mental disorders. In the older age group (> 70 years), disabilities, AFB smear positivity, chronic liver disease, and mental disorders were identified as factors contributing to suicide (Supplementary Table 5, 6, and 7).

In stratified analyses according to the TB treatment phase (during vs. after treatment), male sex, older age, sputum AFB smear positivity, depression, and anxiety disorder consistently showed significant associations with suicide (Supplementary Tables 8 and 9).

## Discussion

In this nationwide study spanning a decade, we investigated the incidence, temporal patterns, and contributing factors of suicide in individuals with TB in Republic of Korea. Over a median follow-up period of 5.5 years, 1314 suicidal deaths were identified, accounting for 1.6% of all recorded deaths. Suicides in men accounted for more than three-quarters of suicidal deaths, and the suicide incidence rate was more than twice as high in men than that in women. Older adults aged ≥ 60 years comprised over half of suicide, with a clear age-dependent increase in suicide incidence. Notably, approximately one-third of suicides occurred during TB treatment, with the highest risk being within the first 30 days of TB diagnosis. Multivariate analyses identified male sex, older age, pulmonary TB, sputum AFB smear positivity, comorbidities, and pre-existing mental disorders as significant risk factors for suicide after TB diagnosis. To the best of our knowledge, this is the first study to comprehensively assess the burden and risk factors for suicide among individuals with TB at a national level over an extended period.

TB is an infectious disease that often requires isolation during the initial contagious phase, which may expose individuals to stigma and social exclusion, contributing to psychological distress [3, 14]. Despite the successful national TB control program and significant decline of TB burden in Republic of Korea, severe stigma associated with TB, particularly among socioeconomically disadvantaged patients with TB, remained underreported and neglected [24]. Notably, perceived discriminatory experiences were significantly related to poor self-rated health in Republic of Korea [25]. Among patients with TB, a high level of stigma was linked to depressive symptoms, whereas social support had an inverse association [26]. Depression is prevalent among patients with TB, with a meta-analysis estimating a pooled prevalence of 45.19% (95% *CI* 38.04–52.55%) [15]. Pathophysiological changes associated with chronic inflammation, such as neuroendocrinological and immunological dysfunction, caused by TB are also thought to contribute to depression and suicide [13]. In addition to the psychological burden, patients with TB frequently experience substantial economic hardship owing to treatment-related direct and indirect medical costs and unemployment during the treatment period [4, 16], with 43% of individuals facing catastrophic costs according to pooled estimates [4]. These combined psychological and economic stressors likely contribute to elevated rates of suicidal ideation and behavior in this population [7].

We found that 411 of 310,194 individuals with TB (0.13%) died of suicide during TB treatment, a rate comparable to those observed in patients with cancer (0.15%) [27], and stroke (0.12%) [28]. These findings support existing evidence that a diagnosis of severe physical illness is a major risk factor for suicide [29]. Importantly, the incidence rate of suicide within one year after TB diagnosis was 2.04/1000 person-years, which was markedly higher than the national average and highlights the vulnerability of patients with TB in the immediate post-diagnosis period [8, 10, 30].

The demographic patterns observed in our study align with national suicide statistics: suicide rates in Republic of Korea are more than twice as high among men compared to that in women and increase with age, particularly among those aged ≥ 70 years [30]. The higher rate of completed suicide among men has been partly attributed to the use of more lethal methods such as fire, jumping from heights, or firearms [31, 32]. In older adults, suicide is often associated with physical decline, loss of independence, and co-existing chronic illnesses [33]. Our stratified analysis revealed that older age was a significant suicide risk factor among men but not women, suggesting that sex-specific biologic or socio-behavioral mechanism may underlie age-related suicide risk in patients with TB. Further studies are required to clarify this association in women.

Notably, suicides were highly concentrated within the first month following TB diagnosis, and the elevated risk persisted throughout the initial 90-day period, suggesting a critical window for early psychological intervention. This pattern has also been reported in patients newly diagnosed with other serious medical conditions [29]. The acute psychosocial impact of a TB diagnosis, particularly when combined with the stigma of infectiousness, as reflected by the higher risk in those with pulmonary TB and smear positivity, may trigger suicidal ideation and behavior. Early psychological support may be critical during this vulnerable period. Unfortunately, no official social support is currently available to patients with TB dealing with isolation or social disconnection, particularly in the early stages following TB diagnosis. In this context, vulnerability assessment at the time of TB diagnosis and enhanced community-based care may be helpful for suicide prevention [34]. The following intervention programs should be considered for suicide prevention: early psychological screening at diagnosis, integration of suicide risk assessment in TB treatment protocols, community-based mental health linkage models, and peer support programs during the first months of treatment.

Despite the strengths of our study, including its large national sample size and robust link between TB and mortality data, several limitations must be acknowledged. First, suicide deaths were identified using national death certificate data, which may underreport suicides, particularly among older adults [35]. Given that the suicides of older adults are more likely to be underreported than those of people of other ages [36], the real suicide rate among men and older adults can be higher than that in our findings. Second, because this was an observational study, the association between several individual characteristics and suicide may not be causal. Third, time-dependent variables such as change in comorbid condition were not modeled dynamically and all covariates used in multivariable models were treated as fixed over time. Furthermore, several potential confounding factors, such as medication side effects, social support, and concurrent psychiatric treatment could not be accounted for. Finally, although our findings are relevant to high-income countries with well-established TB control programs, they may not be generalizable to low-resource settings.

Nevertheless, our findings highlighted the urgent need for integrated mental health support in TB care, particularly during the early treatment phase. Although Republic of Korea has implemented national suicide prevention policies since 2004 [37], the suicide rate remains high, and the stable incidence observed within one year of TB diagnosis suggests that these efforts may not sufficiently reach this vulnerable population. Individuals with TB, particularly newly diagnosed individuals, should be considered a high-risk group for suicide prevention strategies.

Our findings provide evidence for age- and context-specific interventions. Among younger adults (age < 40 years), preexisting mental health conditions are major risk factors, suggesting the need for appropriate psychiatric interventions. In economically active middle-aged adults (age 40–69 years), low income and infectiousness are predominant risk factors, underscoring the importance of socioeconomic support for mitigating stigmatization and providing economic assistance during treatment. For older adults (age > 70 years), comorbidities and disability are key drivers of suicide, indicating the need for multidisciplinary care to address existing chronic health conditions. In addition, considering protective factors against suicide, including perceived control, self-regulation and satisfaction, purpose-in-life, social support and connectedness, sense of belonging, and positive relationship, individualized intervention programs tailored to the contexts of TB should be developed [38].

## Conclusions

Suicide is a significant but underrecognized outcome among individuals with TB. Suicides in men and older adults accounted for more than half of the suicidal deaths, with the highest risk being shortly after diagnosis. A combination of demographic, clinical, socioeconomic, and psychological factors contribute to suicide risk. The incidence of suicide within the first year following a TB diagnosis remained largely unchanged over time, highlighting the urgent need for earlier identification and timely intervention among high-risk individuals. Integrating mental health screening and support into the TB care continuum—through multi-sectoral collaborations across health, social welfare, and mental health systems—is essential to improve patient outcomes and prevent avoidable deaths. It would be valuable for future research to evaluate psychosocial interventions within TB programs and to examine temporal changes in mental health trajectories after TB diagnosis.

## Supplementary Information

Additional file 1

## Data Availability

The release of data by researchers is not legally permitted. All data are available from the database of the National Health Insurance Sharing Service (NHISS) (https://nhiss.nhis.or.kr/bd/ay/bdaya001iv.do). The NHISS allows data to be used at some cost by any researcher who agrees to abide by research ethics. The data for this article can be accessed and downloaded from the website after agreeing to abide by the ethical rules.
