# Supplementary material for: Suicide risk in people with tuberculosis in Republic of Korea: a nationwide cohort, 2012–2021
Source: Infect Dis Poverty. 2026 Mar 23;15:34. doi: 10.1186/s40249-026-01424-x (PMC13007346; doi:10.1186/s40249-026-01424-x)

**Supplementary Table 1.** Diagnostic codes in the 10th revision of the International Classification of Diseases (ICD-10) used to identify comorbidities and mental disorders

| Variables | ICD-10 codes |
| --- | --- |
| Cardiovascular disease | I21, I22, I252, I43, I50, I099, I110, I130, I132, I255, I420, I425, I426, I427, I428, I429, P290, I70, I71, I731, I738, I739, I771, I790, I792, K551, K558, K559, Z958, Z959 |
| Cerebrovascular disease | G45, G46, I60, I61, I62, I63, I64, I65, I66, I67, I68, I69, H340 |
| Dementia | F00, F01, F02, F03, G30, G311, F051 |
| Chronic obstructive pulmonary disease | J40, J41, J42, J43, J44 |
| Rheumatic disease | M05, M06, M32, M33, M34, M315, M351, M353, M360 |
| Peptic ulcer | K25, K26, K27, K28 |
| Chronic liver disease | B18, K73, K74, K700, K701, K702, K703, K709, K713, K714, K715, K717, K760, K762, K763, K764, K768, K769, Z944, I850, I859, I864, I982, K704, K711, K721, K729, K765, K766, K767 |
| Diabetes | E100, E101, E106, E108, E109, E110, E111, E116, E118, E119, E120, E121, E126, E128, E129, E130, E131, E136, E138, E139, E140, E141, E146, E148, E149, E102, E103, E104, E105, E107, E112, E113, E114, E115, E117, E122, E123, E124, E125, E127, E132, E133, E134, E135, E137, E142, E143, E144, E145, E147 |
| Hemiplegia | G81, G82, G041, G114, G801, G802, G830, G831, G832, G833, G834, G839 |
| Chronic kidney disease | N18, N19, I120, I131, N032, N033, N034, N035, N036, N037, N052, N053, N054, N055, N056, N057, N250, Z490, Z491, Z492, Z940, Z992 |
| Malignancy | C00, C01, C02, C03, C04, C05, C06, C07, C08, C09, C10, C11, C12, C13, C14, C15, C16, C17, C18, C19, C20, C21, C22, C23, C24, C25, C26, C30, C31, C32, C33, C34, C37, C38, C39, C40, C41, C43, C45, C46, C47, C48, C49, C50, C51, C52, C53, C54, C55, C56, C57, C58, C60, C61, C62, C63, C64, C65, C66, C67, C68, C69, C70, C71, C72, C73, C74, C75, C76, C81, C82, C83, C84, C85, C88, C90, C91, C92, C93, C94, C95, C96, C97, C77, C78, C79, C80 |
| Living with Human immunodeficiency virus | B20, B21, B22, B24 |
| Depression | F32, F33 |
| Anxiety disorder | F40, F41 |
| Bipolar disorder | F30, F31 |
| Affective disorder | F34 |
| Schizophrenia | F20, F21, F22, F23, F24, F25, F26, F27, F28, F29 |
| Alcohol use disorder | F10 |
| Substance use disorder | F11, F12, F13, F14, F15, F16, F17, F18, F19 |

**Supplementary Table 2.** General characteristics of participants by sex

|  | Total | | Male | | Female | |
| --- | --- | --- | --- | --- | --- | --- |
| *N* | 310,194 (100%) | | 183,277 (59.08%) | | 126,917 (40.92%) | |
|  | *n* | % | *n* | % | *n* | % |
| Age, years | 57.8 ± 20.0 |  | 56.9 ± 18.7 |  | 59.1 ± 21.7 |  |
| 0–19 years | 7686 | 2.48 | 4617 | 2.52 | 3069 | 2.42 |
| 20–29 years | 27,687 | 8.93 | 14,418 | 7.87 | 13,269 | 10.45 |
| 30–39 years | 30,316 | 9.77 | 16,594 | 9.05 | 13,722 | 10.81 |
| 40–49 years | 39,365 | 12.69 | 25,328 | 13.82 | 14,037 | 11.06 |
| 50–59 years | 52,539 | 16.94 | 36,821 | 20.09 | 15,718 | 12.38 |
| 60–69 years | 46,192 | 14.89 | 31,636 | 17.26 | 14,556 | 11.47 |
| 70–79 years | 56,695 | 18.28 | 32,192 | 17.56 | 24,503 | 19.31 |
| ≥ 80 years | 49,714 | 16.03 | 21,671 | 11.82 | 28,043 | 22.10 |
| Region (Province) | 176,247 | 56.82 | 103,005 | 56.20 | 73,242 | 57.71 |
| Low income (Q0) | 25,111 | 8.10 | 14,907 | 8.13 | 10,204 | 8.04 |
| Low income (Q1) | 51,756 | 16.69 | 30,226 | 16.49 | 21,530 | 16.96 |
| Disabilities | 42,645 | 13.75 | 27,596 | 15.06 | 15,049 | 11.86 |
| Pulmonary TB | 246,326 | 79.41 | 151,588 | 82.71 | 94,738 | 74.65 |
| Drug resistance | 13,889 | 4.48 | 8880 | 4.85 | 5009 | 3.95 |
| Previous TB history | 32,856 | 10.59 | 23,291 | 12.71 | 9565 | 7.54 |
| Positive AFB smear | 92,039 | 29.67 | 56,711 | 30.94 | 35,328 | 27.84 |
| CCI score | 2.2 ± 1.9 |  | 2.1 ± 1.9 |  | 2.2 ± 1.9 |  |
| Cardiovascular disease | 63,974 | 20.62 | 35,113 | 19.16 | 28,861 | 22.74 |
| Cerebrovascular disease | 39,604 | 12.77 | 22,228 | 12.13 | 17,376 | 13.69 |
| Dementia | 28,680 | 9.25 | 12,554 | 6.85 | 16,126 | 12.71 |
| COPD | 136,523 | 44.01 | 79,510 | 43.38 | 57,013 | 44.92 |
| Rheumatic disease | 13,332 | 4.30 | 5957 | 3.25 | 7375 | 5.81 |
| Peptic ulcer | 82,073 | 26.46 | 45,591 | 24.88 | 36,482 | 28.74 |
| Chronic liver disease | 92,296 | 29.75 | 57,444 | 31.34 | 34,852 | 27.46 |
| Diabetes | 91,933 | 29.64 | 57,807 | 31.54 | 34,126 | 26.89 |
| Hemiplegia | 5989 | 1.93 | 3698 | 2.02 | 2291 | 1.81 |
| Chronic kidney disease | 13,423 | 4.33 | 8373 | 4.57 | 5050 | 3.98 |
| Malignancy | 35,985 | 11.60 | 23,269 | 12.7 | 12,716 | 10.02 |
| Living with HIV | 594 | 0.19 | 550 | 0.30 | 44 | 0.03 |
| Depression | 37,076 | 11.95 | 19,369 | 10.57 | 17,707 | 13.95 |
| Anxiety disorder | 49,944 | 16.10 | 25,261 | 13.78 | 24,683 | 19.45 |
| Bipolar disorder | 11,659 | 3.76 | 6698 | 3.65 | 4961 | 3.91 |
| Affective disorder | 2009 | 0.65 | 978 | 0.53 | 1031 | 0.81 |
| Schizophrenia | 7112 | 2.29 | 4497 | 2.45 | 2615 | 2.06 |
| Alcohol use disorder | 5755 | 1.86 | 5318 | 2.90 | 437 | 0.34 |
| Substance use disorder | 334 | 0.11 | 237 | 0.13 | 97 | 0.08 |

TB, tuberculosis; AFB, acid-fast bacilli; CCI, Charlson comorbidity index; COPD, chronic obstructive pulmonary disease; HIV, human immunodeficiency virus.

**Supplementary Table 3**. Estimated hazard ratios for demographic, tuberculosis-related, and comorbid risk factors associated with suicide death among male participants

|  | Univariate analysis | | | Multivariate analysis  Model 1 | | | Multivariate analysis  Model 2 | | |
| --- | --- | --- | --- | --- | --- | --- | --- | --- | --- |
|  | HR | 95% *CI* |  | aHR | 95% *CI* |  | aHR | 95% *CI* |  |
| Age, years |  |  |  |  |  |  |  |  |  |
| 0–19 | Ref. |  |  | Ref. |  |  | Ref. |  |  |
| 20–29 | 2.363 | 1.005 | 5.559 | 2.174 | 0.924 | 5.118 | 2.146 | 0.912 | 5.050 |
| 30–39 | 2.963 | 1.279 | 6.862 | 2.572 | 1.109 | 5.963 | 2.517 | 1.085 | 5.836 |
| 40–49 | 4.062 | 1.788 | 9.228 | 3.178 | 1.396 | 7.235 | 3.092 | 1.358 | 7.041 |
| 50–59 | 6.076 | 2.701 | 13.668 | 4.330 | 1.918 | 9.771 | 4.233 | 1.874 | 9.559 |
| 60–69 | 6.053 | 2.681 | 13.666 | 4.102 | 1.808 | 9.307 | 4.050 | 1.784 | 9.195 |
| 70–79 | 9.790 | 4.353 | 22.020 | 6.301 | 2.780 | 14.281 | 6.298 | 2.777 | 14.284 |
| ≥ 80 | 14.08 | 6.221 | 31.866 | 8.756 | 3.833 | 20.002 | 8.978 | 3.928 | 20.522 |
| Region (Province) | 1.155 | 1.019 | 1.309 | 1.052 | 0.928 | 1.194 | 1.051 | 0.927 | 1.193 |
| Low income (Q0) | 1.619 | 1.283 | 2.043 | 1.264 | 0.988 | 1.618 | 1.258 | 0.982 | 1.611 |
| Low income (Q1) | 1.010 | 0.823 | 1.239 | 1.236 | 1.002 | 1.524 | 1.231 | 0.998 | 1.518 |
| Disabilities | 1.845 | 1.584 | 2.149 | 1.272 | 1.083 | 1.494 | 1.301 | 1.106 | 1.531 |
| Pulmonary TB | 1.357 | 1.131 | 1.629 | 1.236 | 0.999 | 1.531 | 1.241 | 1.002 | 1.538 |
| Drug resistance | 1.077 | 0.806 | 1.439 | 1.004 | 0.750 | 1.345 | 1.001 | 0.748 | 1.341 |
| Previous TB history | 1.303 | 1.100 | 1.543 | 1.100 | 0.927 | 1.305 | 1.099 | 0.926 | 1.304 |
| Positive AFB smear | 1.481 | 1.299 | 1.689 | 1.372 | 1.199 | 1.571 | 1.379 | 1.204 | 1.579 |
| CCI score | 1.222 | 1.186 | 1.259 | 1.052 | 1.014 | 1.092 |  |  |  |
| Cardiovascular disease | 1.894 | 1.638 | 2.189 |  |  |  | 1.126 | 0.959 | 1.322 |
| Cerebrovascular disease | 1.608 | 1.337 | 1.933 |  |  |  | 0.787 | 0.642 | 0.965 |
| Dementia | 1.326 | 0.985 | 1.785 |  |  |  |  |  |  |
| COPD | 1.530 | 1.352 | 1.731 |  |  |  | 0.952 | 0.836 | 1.085 |
| Rheumatic disease | 1.953 | 1.485 | 2.570 |  |  |  | 1.366 | 1.034 | 1.805 |
| Peptic ulcer | 1.495 | 1.308 | 1.709 |  |  |  | 1.038 | 0.902 | 1.195 |
| Chronic liver disease | 1.571 | 1.383 | 1.784 |  |  |  | 1.176 | 1.026 | 1.347 |
| Diabetes | 1.536 | 1.350 | 1.748 |  |  |  | 0.994 | 0.864 | 1.144 |
| Hemiplegia | 1.856 | 1.227 | 2.807 |  |  |  | 1.212 | 0.784 | 1.871 |
| Chronic kidney disease | 1.475 | 1.086 | 2.003 |  |  |  | 0.954 | 0.696 | 1.308 |
| Malignancy | 1.610 | 1.348 | 1.923 |  |  |  | 1.193 | 0.994 | 1.432 |
| Living with HIV | 2.212 | 1.053 | 4.648 |  |  |  | 2.614 | 1.238 | 5.521 |
| Depression | 3.033 | 2.609 | 3.525 | 1.765 | 1.479 | 2.105 | 1.798 | 1.507 | 2.146 |
| Anxiety disorder | 2.453 | 2.124 | 2.834 | 1.385 | 1.175 | 1.633 | 1.387 | 1.176 | 1.636 |
| Bipolar disorder | 2.449 | 1.846 | 3.249 | 1.072 | 0.788 | 1.458 | 1.104 | 0.811 | 1.504 |
| Affective disorder | 4.357 | 2.799 | 6.782 | 2.177 | 1.388 | 3.416 | 2.206 | 1.406 | 3.462 |
| Schizophrenia | 2.276 | 1.671 | 3.101 | 1.272 | 0.910 | 1.777 | 1.277 | 0.913 | 1.787 |
| Alcohol use disorder | 1.953 | 1.474 | 2.588 | 1.200 | 0.886 | 1.624 | 1.168 | 0.860 | 1.586 |
| Substance use disorder | 4.212 | 1.750 | 10.139 | 2.336 | 0.965 | 5.656 | 2.289 | 0.945 | 5.544 |

HR, hazard ratio; aHR, adjusted hazard ratio; TB, tuberculosis; AFB, acid-fast bacilli; CCI, Charlson comorbidity index; COPD, chronic obstructive pulmonary disease; HIV, human immunodeficiency virus.

**Supplementary Table 4.** Estimated hazard ratios for demographic, tuberculosis-related, and comorbid risk factors associated with suicide death among female participants

|  | Univariate analysis | | | Multivariate analysis  Model 1 | | | Multivariate analysis  Model 2 | | |
| --- | --- | --- | --- | --- | --- | --- | --- | --- | --- |
|  | HR | 95% *CI* |  | aHR | 95% *CI* |  | aHR | 95% *CI* |  |
| Age, years |  |  |  |  |  |  |  |  |  |
| 0–19 | Ref. |  |  | Ref. |  |  | Ref. |  |  |
| 20–29 | 0.616 | 0.285 | 1.332 | 0.574 | 0.265 | 1.242 | 0.566 | 0.261 | 1.227 |
| 30–39 | 0.927 | 0.446 | 1.929 | 0.807 | 0.387 | 1.686 | 0.791 | 0.379 | 1.653 |
| 40–49 | 0.888 | 0.425 | 1.856 | 0.739 | 0.351 | 1.552 | 0.715 | 0.340 | 1.504 |
| 50–59 | 0.654 | 0.306 | 1.396 | 0.493 | 0.228 | 1.065 | 0.467 | 0.216 | 1.009 |
| 60–69 | 1.030 | 0.495 | 2.144 | 0.687 | 0.324 | 1.458 | 0.638 | 0.301 | 1.354 |
| 70–79 | 1.318 | 0.658 | 2.642 | 0.775 | 0.374 | 1.605 | 0.722 | 0.349 | 1.493 |
| ≥ 80 | 1.832 | 0.914 | 3.669 | 1.072 | 0.517 | 2.221 | 1.017 | 0.492 | 2.103 |
| Region (Province) | 1.336 | 1.058 | 1.686 | 1.197 | 0.945 | 1.516 | 1.199 | 0.947 | 1.519 |
| Low income (Q0) | 1.190 | 0.750 | 1.887 | 0.859 | 0.536 | 1.376 | 0.842 | 0.525 | 1.349 |
| Low income (Q1) | 1.193 | 0.846 | 1.681 | 1.324 | 0.936 | 1.872 | 1.319 | 0.933 | 1.865 |
| Disabilities | 1.526 | 1.105 | 2.108 | 1.088 | 0.775 | 1.529 | 1.085 | 0.773 | 1.523 |
| Pulmonary TB | 1.396 | 1.053 | 1.852 | 1.190 | 0.833 | 1.701 | 1.201 | 0.840 | 1.716 |
| Drug resistance | 1.483 | 0.896 | 2.454 | 1.364 | 0.820 | 2.268 | 1.368 | 0.822 | 2.275 |
| Previous TB history | 1.385 | 0.965 | 1.988 | 1.424 | 0.988 | 2.053 | 1.427 | 0.990 | 2.058 |
| Positive AFB smear | 1.249 | 0.973 | 1.604 | 1.111 | 0.858 | 1.439 | 1.106 | 0.854 | 1.433 |
| CCI score | 1.168 | 1.104 | 1.236 | 1.021 | 0.950 | 1.098 |  |  |  |
| Cardiovascular disease | 1.564 | 1.206 | 2.028 |  |  |  | 0.956 | 0.714 | 1.281 |
| Cerebrovascular disease | 1.749 | 1.293 | 2.366 |  |  |  | 1.012 | 0.728 | 1.406 |
| Dementia | 1.406 | 0.967 | 2.045 |  |  |  |  |  |  |
| COPD | 1.059 | 0.845 | 1.329 |  |  |  |  |  |  |
| Rheumatic disease | 1.433 | 0.937 | 2.193 |  |  |  |  |  |  |
| Peptic ulcer | 1.478 | 1.170 | 1.868 |  |  |  | 1.138 | 0.889 | 1.456 |
| Chronic liver disease | 1.476 | 1.162 | 1.874 |  |  |  | 1.166 | 0.907 | 1.499 |
| Diabetes | 1.659 | 1.303 | 2.112 |  |  |  | 1.178 | 0.898 | 1.544 |
| Hemiplegia | 1.152 | 0.429 | 3.093 |  |  |  |  |  |  |
| Chronic kidney disease | 1.270 | 0.675 | 2.386 |  |  |  |  |  |  |
| Malignancy | 0.928 | 0.616 | 1.397 |  |  |  |  |  |  |
| Living with HIV | 0 | 0 | ∞ |  |  |  |  |  |  |
| Depression | 3.284 | 2.571 | 4.193 | 2.149 | 1.614 | 2.862 | 2.119 | 1.593 | 2.819 |
| Anxiety disorder | 2.557 | 2.021 | 3.234 | 1.668 | 1.274 | 2.184 | 1.627 | 1.242 | 2.132 |
| Bipolar disorder | 2.941 | 1.865 | 4.637 | 1.116 | 0.679 | 1.836 | 1.119 | 0.681 | 1.841 |
| Affective disorder | 3.099 | 1.465 | 6.557 | 1.619 | 0.758 | 3.460 | 1.600 | 0.749 | 3.419 |
| Schizophrenia | 4.915 | 3.184 | 7.589 | 3.057 | 1.894 | 4.935 | 3.062 | 1.897 | 4.942 |
| Alcohol use disorder | 3.032 | 0.973 | 9.454 |  |  |  |  |  |  |
| Substance use disorder | 5.142 | 0.724 | 36.525 |  |  |  |  |  |  |

HR, hazard ratio; aHR, adjusted hazard ratio; TB, tuberculosis; AFB, acid-fast bacilli; CCI, Charlson comorbidity index; COPD, chronic obstructive pulmonary disease; HIV, human immunodeficiency virus.

**Supplementary Table 5**. Estimated hazard ratios for demographic, tuberculosis-related, and comorbid risk factors associated with suicide death among participants aged < 40 years

|  | Univariate analysis | | | Multivariate analysis  Model 1 | | | Multivariate analysis  Model 2 | | |
| --- | --- | --- | --- | --- | --- | --- | --- | --- | --- |
|  | HR | 95% *CI* |  | aHR | 95% *CI* |  | aHR | 95% *CI* |  |
| Male sex | 1.352 | 0.996 | 1.835 | 1.397 | 1.025 | 1.905 | 1.370 | 1.004 | 1.869 |
| Region (Province) | 0.995 | 0.739 | 1.339 | 0.963 | 0.714 | 1.298 | 0.965 | 0.716 | 1.301 |
| Low income (Q0) | 1.805 | 0.846 | 3.853 | 0.849 | 0.372 | 1.940 | 0.838 | 0.366 | 1.916 |
| Low income (Q1) | 1.331 | 0.801 | 2.210 | 1.248 | 0.750 | 2.077 | 1.240 | 0.745 | 2.065 |
| Disabilities | 2.776 | 1.544 | 4.989 | 1.236 | 0.617 | 2.476 | 1.262 | 0.630 | 2.525 |
| Pulmonary TB | 1.295 | 0.869 | 1.929 | 1.274 | 0.788 | 2.058 | 1.309 | 0.809 | 2.117 |
| Drug resistance | 1.771 | 0.961 | 3.264 | 1.590 | 0.854 | 2.962 | 1.571 | 0.843 | 2.928 |
| Previous TB history | 1.802 | 1.119 | 2.904 | 1.575 | 0.971 | 2.554 | 1.567 | 0.966 | 2.542 |
| Positive AFB smear | 1.140 | 0.805 | 1.613 | 1.024 | 0.717 | 1.462 | 1.024 | 0.717 | 1.462 |
| CCI score | 1.308 | 1.142 | 1.499 | 1.160 | 1.008 | 1.336 |  |  |  |
| Cardiovascular disease | 1.538 | 0.571 | 4.146 |  |  |  |  |  |  |
| Cerebrovascular disease | 2.091 | 0.519 | 8.422 |  |  |  |  |  |  |
| Dementia | 0 | 0 | ∞ |  |  |  |  |  |  |
| COPD | 1.020 | 0.731 | 1.422 |  |  |  |  |  |  |
| Rheumatic disease | 2.527 | 1.186 | 5.382 |  |  |  | 2.069 | 0.960 | 4.460 |
| Peptic ulcer | 1.540 | 1.054 | 2.250 |  |  |  | 1.264 | 0.855 | 1.869 |
| Chronic liver disease | 1.596 | 1.114 | 2.286 |  |  |  | 1.110 | 0.750 | 1.642 |
| Diabetes | 2.475 | 1.519 | 4.032 |  |  |  | 1.638 | 0.971 | 2.765 |
| Hemiplegia | 0 | 0 | ∞ |  |  |  |  |  |  |
| Chronic kidney disease | 1.231 | 0.172 | 8.787 |  |  |  |  |  |  |
| Malignancy | 1.632 | 0.671 | 3.973 |  |  |  |  |  |  |
| Living with HIV | 5.726 | 1.421 | 23.073 |  |  |  | 3.093 | 0.739 | 12.945 |
| Depression | 6.826 | 4.629 | 10.064 | 3.897 | 2.357 | 6.444 | 3.831 | 2.324 | 6.316 |
| Anxiety disorder | 3.106 | 2.019 | 4.779 | 1.196 | 0.701 | 2.043 | 1.185 | 0.695 | 2.022 |
| Bipolar disorder | 9.836 | 5.470 | 17.686 | 1.559 | 0.732 | 3.321 | 1.525 | 0.713 | 3.259 |
| Affective disorder | 2.640 | 0.370 | 18.845 |  |  |  |  |  |  |
| Schizophrenia | 11.868 | 6.989 | 20.152 | 3.805 | 1.884 | 7.688 | 3.879 | 1.915 | 7.859 |
| Alcohol use disorder | 7.033 | 3.116 | 15.877 | 1.337 | 0.538 | 3.321 | 1.257 | 0.499 | 3.166 |
| Substance use disorder | 12.425 | 1.740 | 88.702 | 1.555 | 0.201 | 12.012 | 1.346 | 0.168 | 10.802 |

HR, hazard ratio; aHR, adjusted hazard ratio; TB, tuberculosis; AFB, acid-fast bacilli; CCI, Charlson comorbidity index; COPD, chronic obstructive pulmonary disease; HIV, human immunodeficiency virus.

**Supplementary Table 6**. Estimated hazard ratios for demographic, tuberculosis-related, and comorbid risk factors associated with suicide death among participants aged 40–69 years

|  | Univariate analysis | | | Multivariate analysis  Model 1 | | | Multivariate analysis  Model 2 | | |
| --- | --- | --- | --- | --- | --- | --- | --- | --- | --- |
|  | HR | 95% *CI* |  | aHR | 95% *CI* |  | aHR | 95% *CI* |  |
| Male sex | 2.806 | 2.252 | 3.497 | 2.674 | 2.133 | 3.354 | 2.646 | 2.108 | 3.320 |
| Region (Province) | 1.204 | 1.025 | 1.415 | 1.168 | 0.993 | 1.372 | 1.169 | 0.994 | 1.373 |
| Low income (Q0) | 2.844 | 2.081 | 3.886 | 1.638 | 1.166 | 2.300 | 1.618 | 1.152 | 2.274 |
| Low income (Q1) | 1.692 | 1.269 | 2.257 | 1.490 | 1.116 | 1.990 | 1.482 | 1.110 | 1.979 |
| Disabilities | 1.490 | 1.200 | 1.850 | 1.049 | 0.829 | 1.329 | 1.050 | 0.828 | 1.332 |
| Pulmonary TB | 1.662 | 1.320 | 2.093 | 1.217 | 0.917 | 1.615 | 1.230 | 0.927 | 1.634 |
| Drug resistance | 1.013 | 0.688 | 1.490 | 0.847 | 0.574 | 1.250 | 0.849 | 0.575 | 1.253 |
| Previous TB history | 1.309 | 1.058 | 1.620 | 1.156 | 0.933 | 1.433 | 1.161 | 0.936 | 1.439 |
| Positive AFB smear | 1.489 | 1.255 | 1.766 | 1.390 | 1.167 | 1.656 | 1.391 | 1.167 | 1.657 |
| CCI score | 1.142 | 1.092 | 1.195 | 1.068 | 1.017 | 1.121 |  |  |  |
| Cardiovascular disease | 1.502 | 1.220 | 1.849 |  |  |  | 1.226 | 0.985 | 1.527 |
| Cerebrovascular disease | 1.127 | 0.829 | 1.532 |  |  |  |  |  |  |
| Dementia | 1.139 | 0.627 | 2.069 |  |  |  |  |  |  |
| COPD | 1.162 | 0.990 | 1.365 |  |  |  |  |  |  |
| Rheumatic disease | 1.325 | 0.923 | 1.902 |  |  |  |  |  |  |
| Peptic ulcer | 1.076 | 0.898 | 1.289 |  |  |  |  |  |  |
| Chronic liver disease | 1.440 | 1.222 | 1.697 |  |  |  | 1.167 | 0.979 | 1.391 |
| Diabetes | 1.384 | 1.170 | 1.637 |  |  |  | 1.017 | 0.849 | 1.217 |
| Hemiplegia | 2.268 | 1.359 | 3.787 |  |  |  | 1.462 | 0.863 | 2.479 |
| Chronic kidney disease | 1.230 | 0.787 | 1.920 |  |  |  |  |  |  |
| Malignancy | 1.144 | 0.889 | 1.472 |  |  |  |  |  |  |
| Living with HIV | 2.365 | 0.885 | 6.321 |  |  |  |  |  |  |
| Depression | 2.553 | 2.090 | 3.120 | 1.861 | 1.463 | 2.366 | 1.871 | 1.473 | 2.377 |
| Anxiety disorder | 1.939 | 1.601 | 2.349 | 1.458 | 1.169 | 1.819 | 1.470 | 1.181 | 1.831 |
| Bipolar disorder | 2.612 | 1.787 | 3.818 | 1.089 | 0.713 | 1.663 | 1.089 | 0.713 | 1.663 |
| Affective disorder | 3.777 | 2.081 | 6.858 | 2.293 | 1.247 | 4.216 | 2.297 | 1.249 | 4.224 |
| Schizophrenia | 2.703 | 1.912 | 3.820 | 1.527 | 1.032 | 2.260 | 1.523 | 1.030 | 2.251 |
| Alcohol use disorder | 2.516 | 1.839 | 3.443 | 1.016 | 0.718 | 1.438 | 0.995 | 0.701 | 1.412 |
| Substance use disorder | 5.584 | 2.089 | 14.927 | 2.647 | 0.979 | 7.161 | 2.665 | 0.985 | 7.208 |

HR, hazard ratio; aHR, adjusted hazard ratio; TB, tuberculosis; AFB, acid-fast bacilli; CCI, Charlson comorbidity index; COPD, chronic obstructive pulmonary disease; HIV, human immunodeficiency virus.

**Supplementary Table 7**. Estimated hazard ratios for demographic, tuberculosis-related, and comorbid risk factors associated with suicide death among participants aged ≥ 70 years

|  | Univariate analysis | | | Multivariate analysis  Model 1 | | | Multivariate analysis  Model 2 | | |
| --- | --- | --- | --- | --- | --- | --- | --- | --- | --- |
|  | HR | 95% *CI* |  | aHR | 95% *CI* |  | aHR | 95% *CI* |  |
| Male sex | 3.039 | 2.508 | 3.682 | 3.176 | 2.612 | 3.862 | 3.073 | 2.526 | 3.739 |
| Region (Province) | 1.014 | 0.849 | 1.211 | 1.023 | 0.856 | 1.222 | 1.026 | 0.859 | 1.226 |
| Low income (Q0) | 0.840 | 0.596 | 1.183 | 0.840 | 0.595 | 1.185 | 0.857 | 0.607 | 1.209 |
| Low income (Q1) | 0.970 | 0.733 | 1.283 | 1.055 | 0.797 | 1.396 | 1.051 | 0.794 | 1.391 |
| Disabilities | 1.453 | 1.199 | 1.761 | 1.323 | 1.089 | 1.607 | 1.361 | 1.121 | 1.652 |
| Pulmonary TB | 1.396 | 1.101 | 1.771 | 1.220 | 0.923 | 1.612 | 1.213 | 0.918 | 1.604 |
| Drug resistance | 1.318 | 0.889 | 1.956 | 1.227 | 0.825 | 1.824 | 1.228 | 0.826 | 1.826 |
| Previous TB history | 1.340 | 1.045 | 1.718 | 1.043 | 0.811 | 1.342 | 1.025 | 0.797 | 1.318 |
| Positive AFB smear | 1.253 | 1.047 | 1.500 | 1.304 | 1.083 | 1.570 | 1.292 | 1.073 | 1.556 |
| CCI score | 1.077 | 1.031 | 1.125 | 1.031 | 0.985 | 1.079 |  |  |  |
| Cardiovascular disease | 1.105 | 0.928 | 1.315 |  |  |  |  |  |  |
| Cerebrovascular disease | 1.110 | 0.913 | 1.350 |  |  |  |  |  |  |
| Dementia | 0.671 | 0.517 | 0.869 |  |  |  | 0.622 | 0.476 | 0.813 |
| COPD | 1.183 | 0.992 | 1.410 |  |  |  |  |  |  |
| Rheumatic disease | 1.196 | 0.864 | 1.658 |  |  |  |  |  |  |
| Peptic ulcer | 1.269 | 1.069 | 1.507 |  |  |  | 1.150 | 0.965 | 1.370 |
| Chronic liver disease | 1.328 | 1.118 | 1.579 |  |  |  | 1.197 | 1.004 | 1.426 |
| Diabetes | 1.110 | 0.935 | 1.317 |  |  |  |  |  |  |
| Hemiplegia | 0.911 | 0.514 | 1.616 |  |  |  |  |  |  |
| Chronic kidney disease | 1.112 | 0.777 | 1.591 |  |  |  |  |  |  |
| Malignancy | 1.218 | 0.976 | 1.522 |  |  |  |  |  |  |
| Living with HIV | 5.103 | 0.720 | 36.183 |  |  |  |  |  |  |
| Depression | 1.865 | 1.549 | 2.245 | 1.686 | 1.379 | 2.060 | 1.818 | 1.486 | 2.225 |
| Anxiety disorder | 1.617 | 1.355 | 1.929 | 1.460 | 1.206 | 1.768 | 1.465 | 1.210 | 1.772 |
| Bipolar disorder | 1.293 | 0.899 | 1.862 |  |  |  |  |  |  |
| Affective disorder | 2.512 | 1.503 | 4.197 | 2.007 | 1.195 | 3.371 | 2.003 | 1.192 | 3.365 |
| Schizophrenia | 1.424 | 0.852 | 2.381 |  |  |  |  |  |  |
| Alcohol use disorder | 1.716 | 0.768 | 3.838 |  |  |  |  |  |  |
| Substance use disorder | 1.866 | 0.262 | 13.267 |  |  |  |  |  |  |

HR, hazard ratio; aHR, adjusted hazard ratio; TB, tuberculosis; AFB, acid-fast bacilli; CCI, Charlson comorbidity index; COPD, chronic obstructive pulmonary disease; HIV, human immunodeficiency virus.

**Supplementary Table 8**. Estimated hazard ratios for demographic, tuberculosis-related, and comorbid risk factors associated with suicide death during tuberculosis treatment

|  | Univariate analysis | | | Multivariate analysis  Model 1 | | | Multivariate analysis  Model 2 | | |
| --- | --- | --- | --- | --- | --- | --- | --- | --- | --- |
|  | HR | 95% *CI* |  | aHR | 95% *CI* |  | aHR | 95% *CI* |  |
| Male sex | 2.130 | 1.694 | 2.679 | 2.618 | 2.066 | 3.317 | 2.637 | 2.078 | 3.346 |
| Age, years |  |  |  |  |  |  |  |  |  |
| 0–19 | Ref. |  |  | Ref. |  |  | Ref. |  |  |
| 20–29 | 2.488 | 0.317 | 19.547 | 2.455 | 0.311 | 19.388 | 2.403 | 0.304 | 18.982 |
| 30–39 | 2.532 | 0.326 | 19.692 | 2.284 | 0.292 | 17.858 | 2.194 | 0.280 | 17.160 |
| 40–49 | 6.108 | 0.838 | 44.535 | 4.772 | 0.650 | 35.02 | 4.455 | 0.607 | 32.708 |
| 50–59 | 7.479 | 1.038 | 53.883 | 5.095 | 0.702 | 36.998 | 4.657 | 0.641 | 33.849 |
| 60–69 | 10.074 | 1.402 | 72.387 | 6.083 | 0.838 | 44.148 | 5.570 | 0.767 | 40.475 |
| 70–79 | 17.148 | 2.407 | 122.19 | 9.748 | 1.350 | 70.373 | 9.210 | 1.275 | 66.553 |
| ≥ 80 | 22.296 | 3.129 | 158.85 | 13.602 | 1.882 | 98.315 | 13.54 | 1.872 | 97.924 |
| Region (Province) | 1.287 | 1.049 | 1.578 | 1.108 | 0.902 | 1.362 | 1.109 | 0.902 | 1.363 |
| Low income (Q0) | 1.260 | 0.876 | 1.811 | 1.081 | 0.744 | 1.570 | 1.074 | 0.739 | 1.561 |
| Low income (Q1) | 0.774 | 0.557 | 1.075 | 1.040 | 0.745 | 1.451 | 1.037 | 0.743 | 1.447 |
| Disabilities | 2.189 | 1.740 | 2.753 | 1.348 | 1.062 | 1.712 | 1.414 | 1.112 | 1.799 |
| Pulmonary TB | 1.353 | 1.035 | 1.768 | 0.987 | 0.716 | 1.360 | 0.968 | 0.702 | 1.334 |
| Drug resistance | 2.578 | 1.878 | 3.539 | 2.472 | 1.793 | 3.408 | 2.468 | 1.79 | 3.404 |
| Previous TB history | 1.199 | 0.889 | 1.618 | 0.939 | 0.693 | 1.272 | 0.934 | 0.689 | 1.266 |
| Positive AFB smear | 1.519 | 1.233 | 1.871 | 1.392 | 1.120 | 1.730 | 1.369 | 1.101 | 1.703 |
| CCI score | 1.270 | 1.216 | 1.326 | 1.051 | 0.996 | 1.110 |  |  |  |
| Cardiovascular disease | 1.936 | 1.565 | 2.395 |  |  |  | 0.936 | 0.741 | 1.182 |
| Cerebrovascular disease | 1.738 | 1.347 | 2.241 |  |  |  | 0.761 | 0.576 | 1.005 |
| Dementia | 1.352 | 0.975 | 1.876 |  |  |  |  |  |  |
| COPD | 1.712 | 1.402 | 2.090 |  |  |  | 0.948 | 0.769 | 1.168 |
| Rheumatic disease | 1.951 | 1.352 | 2.816 |  |  |  | 1.402 | 0.965 | 2.036 |
| Peptic ulcer | 1.898 | 1.551 | 2.323 |  |  |  | 1.277 | 1.033 | 1.578 |
| Chronic liver disease | 1.717 | 1.405 | 2.099 |  |  |  | 1.156 | 0.935 | 1.430 |
| Diabetes | 2.162 | 1.773 | 2.636 |  |  |  | 1.270 | 1.025 | 1.573 |
| Hemiplegia | 1.551 | 0.828 | 2.907 |  |  |  | 0.878 | 0.457 | 1.687 |
| Chronic kidney disease | 1.434 | 0.924 | 2.227 |  |  |  | 0.773 | 0.492 | 1.214 |
| Malignancy | 1.647 | 1.265 | 2.146 |  |  |  | 1.082 | 0.825 | 1.418 |
| Living with HIV | 1.332 | 0.187 | 9.473 |  |  |  | 1.457 | 0.204 | 10.424 |
| Depression | 3.282 | 2.642 | 4.076 | 1.853 | 1.444 | 2.378 | 1.894 | 1.476 | 2.431 |
| Anxiety disorder | 2.907 | 2.362 | 3.577 | 1.710 | 1.353 | 2.161 | 1.681 | 1.328 | 2.127 |
| Bipolar disorder | 2.694 | 1.876 | 3.868 | 1.149 | 0.778 | 1.698 | 1.231 | 0.832 | 1.821 |
| Affective disorder | 5.794 | 3.399 | 9.878 | 3.045 | 1.768 | 5.243 | 3.068 | 1.781 | 5.285 |
| Schizophrenia | 1.985 | 1.203 | 3.273 | 1.026 | 0.606 | 1.739 | 1.041 | 0.613 | 1.766 |
| Alcohol use disorder | 0.836 | 0.373 | 1.872 | 0.463 | 0.203 | 1.058 | 0.436 | 0.191 | 0.998 |
| Substance use disorder | 4.926 | 1.228 | 19.765 | 2.588 | 0.642 | 10.438 | 2.507 | 0.621 | 10.114 |

HR, hazard ratio; aHR, adjusted hazard ratio; TB, tuberculosis; AFB, acid-fast bacilli; CCI, Charlson comorbidity index; COPD, chronic obstructive pulmonary disease; HIV, human immunodeficiency virus.

**Supplementary Table 9**. Estimated hazard ratios for demographic, tuberculosis-related, and comorbid risk factors associated with suicide death after tuberculosis treatment completion

|  | Univariate analysis | | | Multivariate analysis  Model 1 | | | Multivariate analysis  Model 2 | | |
| --- | --- | --- | --- | --- | --- | --- | --- | --- | --- |
|  | HR | 95% *CI* |  | aHR | 95% *CI* |  | aHR | 95% *CI* |  |
| Male sex | 2.525 | 2.163 | 2.948 | 2.660 | 2.265 | 3.124 | 2.645 | 2.251 | 3.108 |
| Age, years |  |  |  |  |  |  |  |  |  |
| 0–19 | Ref. |  |  | Ref. |  |  | Ref. |  |  |
| 20–29 | 1.180 | 0.657 | 2.120 | 1.173 | 0.652 | 2.108 | 1.164 | 0.647 | 2.092 |
| 30–39 | 1.668 | 0.947 | 2.937 | 1.538 | 0.872 | 2.713 | 1.520 | 0.862 | 2.682 |
| 40–49 | 1.929 | 1.109 | 3.357 | 1.496 | 0.857 | 2.611 | 1.477 | 0.846 | 2.580 |
| 50–59 | 2.781 | 1.618 | 4.779 | 1.912 | 1.106 | 3.304 | 1.908 | 1.103 | 3.299 |
| 60–69 | 2.651 | 1.532 | 4.587 | 1.811 | 1.038 | 3.160 | 1.830 | 1.048 | 3.196 |
| 70–79 | 3.212 | 1.866 | 5.529 | 2.386 | 1.368 | 4.161 | 2.439 | 1.397 | 4.258 |
| ≥ 80 | 3.550 | 2.033 | 6.200 | 2.933 | 1.654 | 5.202 | 3.025 | 1.704 | 5.368 |
| Region (Province) | 1.143 | 1.003 | 1.304 | 1.067 | 0.935 | 1.218 | 1.068 | 0.936 | 1.219 |
| Low income (Q0) | 1.730 | 1.344 | 2.226 | 1.172 | 0.897 | 1.533 | 1.164 | 0.890 | 1.523 |
| Low income (Q1) | 1.240 | 1.005 | 1.530 | 1.338 | 1.081 | 1.657 | 1.336 | 1.078 | 1.654 |
| Disabilities | 1.719 | 1.447 | 2.044 | 1.166 | 0.970 | 1.401 | 1.173 | 0.974 | 1.413 |
| Pulmonary TB | 1.588 | 1.319 | 1.913 | 1.351 | 1.081 | 1.688 | 1.376 | 1.100 | 1.721 |
| Drug resistance | 0.578 | 0.375 | 0.891 | 0.502 | 0.325 | 0.776 | 0.502 | 0.325 | 0.776 |
| Previous TB history | 1.540 | 1.289 | 1.840 | 1.275 | 1.065 | 1.528 | 1.282 | 1.070 | 1.536 |
| Positive AFB smear | 1.400 | 1.217 | 1.610 | 1.276 | 1.106 | 1.473 | 1.291 | 1.118 | 1.490 |
| CCI score | 1.163 | 1.125 | 1.203 | 1.038 | 0.996 | 1.082 |  |  |  |
| Cardiovascular disease | 1.614 | 1.378 | 1.891 |  |  |  | 1.157 | 0.970 | 1.380 |
| Cerebrovascular disease | 1.493 | 1.222 | 1.824 |  |  |  | 0.899 | 0.720 | 1.122 |
| Dementia | 1.000 | 0.719 | 1.391 |  |  |  |  |  |  |
| COPD | 1.249 | 1.097 | 1.422 |  |  |  | 1.042 | 0.909 | 1.195 |
| Rheumatic disease | 1.344 | 0.999 | 1.808 |  |  |  | 1.202 | 0.889 | 1.625 |
| Peptic ulcer | 1.237 | 1.073 | 1.427 |  |  |  | 0.982 | 0.844 | 1.142 |
| Chronic liver disease | 1.541 | 1.346 | 1.765 |  |  |  | 1.192 | 1.031 | 1.379 |
| Diabetes | 1.427 | 1.240 | 1.643 |  |  |  | 0.925 | 0.793 | 1.079 |
| Hemiplegia | 1.864 | 1.153 | 3.012 |  |  |  | 1.209 | 0.732 | 1.997 |
| Chronic kidney disease | 1.489 | 1.046 | 2.119 |  |  |  | 1.100 | 0.765 | 1.582 |
| Malignancy | 1.371 | 1.116 | 1.684 |  |  |  | 1.150 | 0.931 | 1.421 |
| Living with HIV | 3.275 | 1.469 | 7.301 |  |  |  | 2.720 | 1.214 | 6.098 |
| Depression | 2.661 | 2.271 | 3.118 | 1.856 | 1.539 | 2.238 | 1.872 | 1.551 | 2.258 |
| Anxiety disorder | 1.951 | 1.674 | 2.273 | 1.329 | 1.116 | 1.584 | 1.332 | 1.117 | 1.588 |
| Bipolar disorder | 2.422 | 1.756 | 3.339 | 1.001 | 0.704 | 1.424 | 1.008 | 0.708 | 1.434 |
| Affective disorder | 2.526 | 1.461 | 4.366 | 1.466 | 0.842 | 2.553 | 1.479 | 0.849 | 2.576 |
| Schizophrenia | 3.410 | 2.550 | 4.560 | 1.967 | 1.422 | 2.722 | 1.975 | 1.427 | 2.733 |
| Alcohol use disorder | 3.348 | 2.504 | 4.477 | 1.479 | 1.076 | 2.032 | 1.452 | 1.054 | 2.001 |
| Substance use disorder | 4.737 | 1.774 | 12.648 | 2.210 | 0.822 | 5.941 | 2.171 | 0.807 | 5.839 |

HR, hazard ratio; aHR, adjusted hazard ratio; TB, tuberculosis; AFB, acid-fast bacilli; CCI, Charlson comorbidity index; COPD, chronic obstructive pulmonary disease; HIV, human immunodeficiency virus.

**Supplementary Figure 1.** Cumulative incidence of suicide death since tuberculosis diagnosis stratified by sex and age, plotted using the Kaplan–Meier survival curve

(A) Within one year after tuberculosis diagnosis and stratified by sex, and (B) Within one year after tuberculosis diagnosis and stratified by age.

(A)


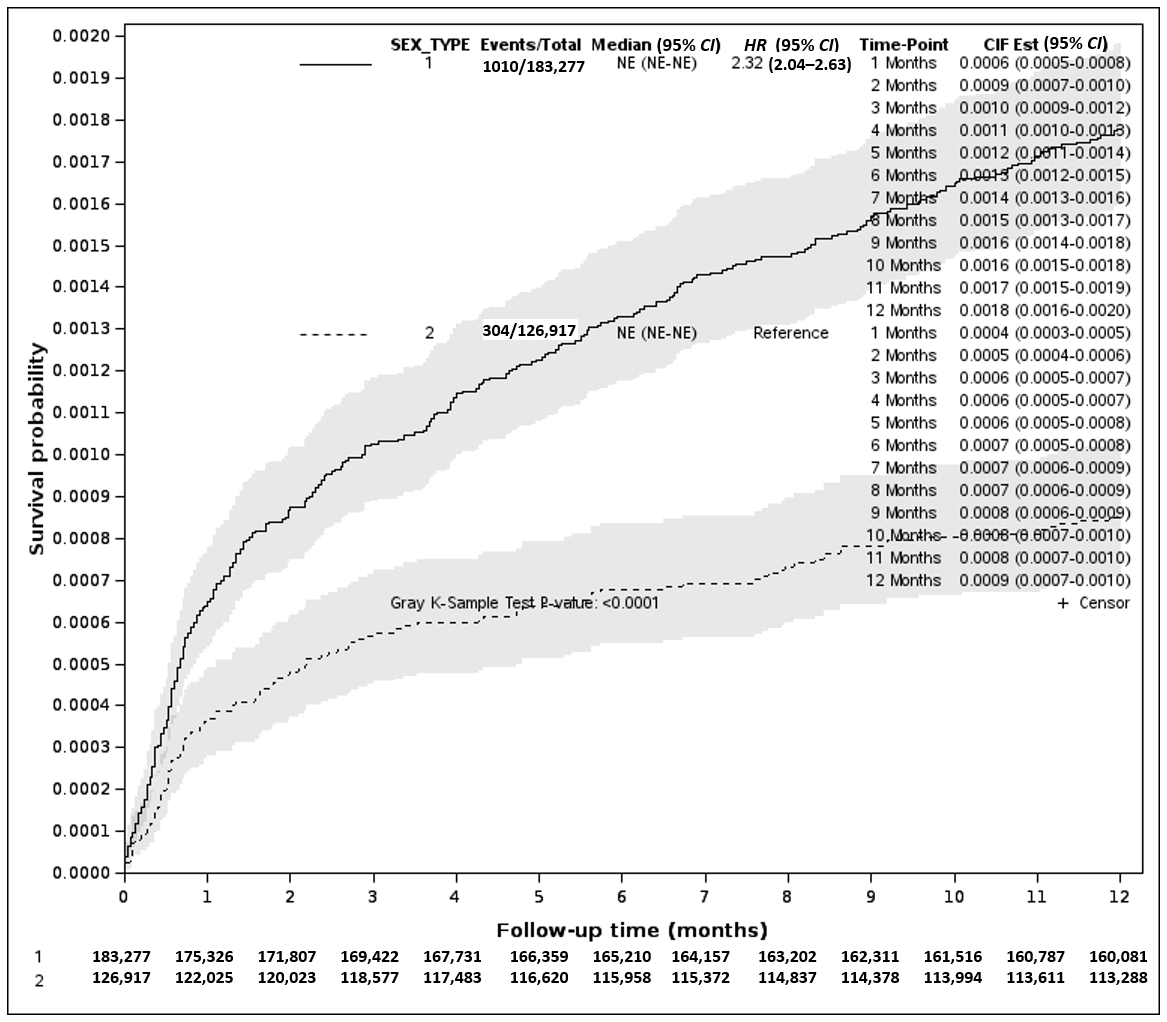


(B)


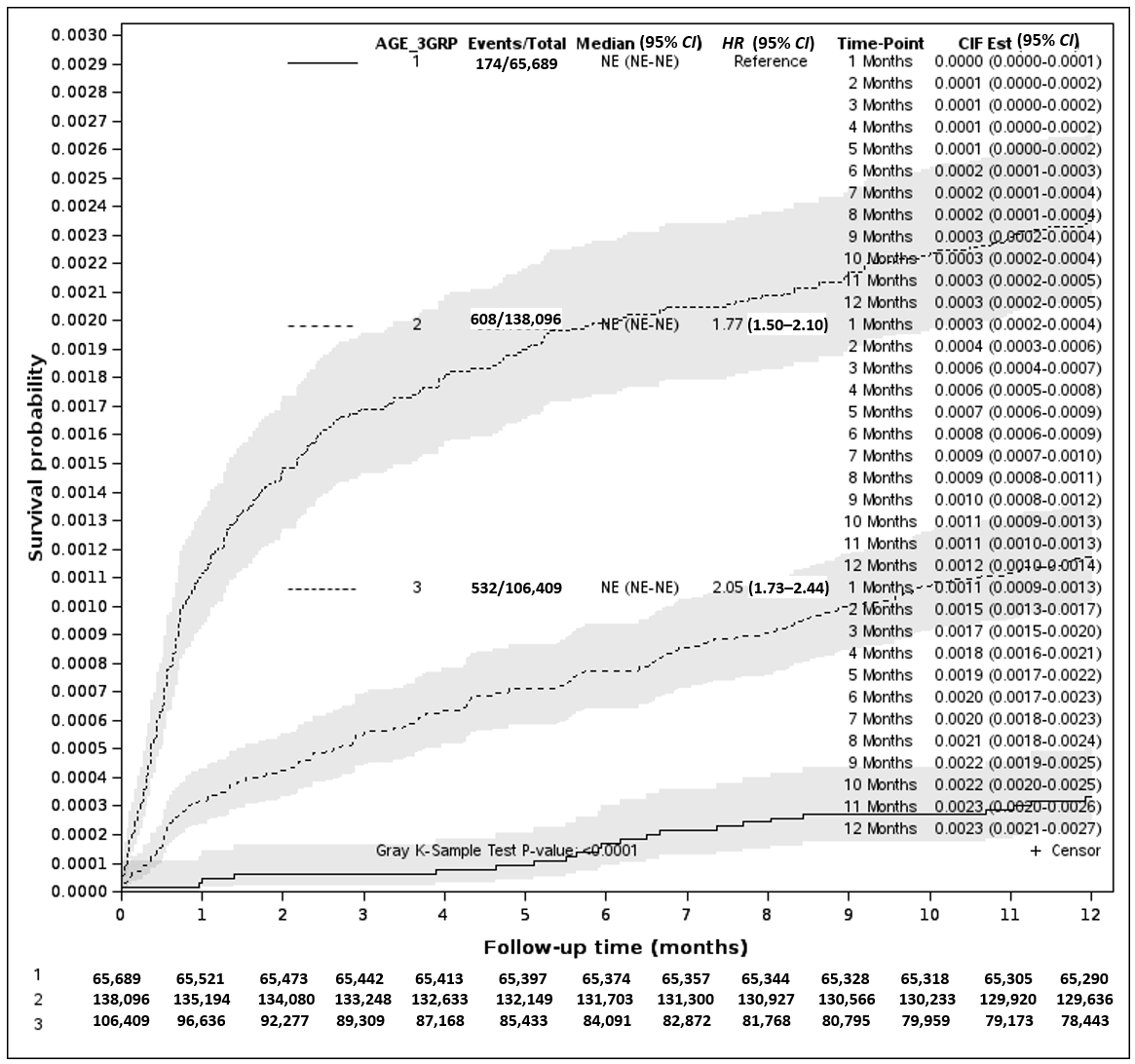

Supplement: Supplementary file 1 — Additional file 1 [file 40249_2026_1424_MOESM1_ESM.docx]
